# Biodegradation of imidazolium ionic liquids by activated sludge microorganisms

**DOI:** 10.1007/s10532-015-9747-0

**Published:** 2015-10-13

**Authors:** Ewa Liwarska-Bizukojc, Cedric Maton, Christian V. Stevens

**Affiliations:** Institute of Fermentation Technology and Microbiology, Lodz University of Technology, ul. Wolczanska 171/173, 90-924 Lodz, Poland; Faculty of Bioscience Engineering, Department of Sustainable Organic Chemistry and Technology, Ghent University, Coupure Links 653, 9000 Ghent, Belgium

**Keywords:** Activated sludge, Biodegradation, Imidazolium ionic liquids, Intermediate metabolites, Mass spectrometry

## Abstract

Biological properties of ionic liquids (ILs) have been usually tested with the help of standard biodegradation or ecotoxicity tests. So far, several articles on the identification of intermediate metabolites of microbiological decay of ILs have been published. Simultaneously, the number of novel ILs with unrecognized characteristics regarding biodegradability and effect on organisms and environment is still increasing. In this work, seven imidazolium ionic liquids of different chemical structure were studied. Three of them are 1-alkyl-3-methyl-imidazolium bromides, while the other four are tetra- or completely substituted imidazolium iodides. This study focused on the identification of intermediate metabolites of the aforementioned ionic liquids subjected to biodegradation in a laboratory activated sludge system. Both fully substituted ionic liquids and 1-ethyl-3-methyl-imidazolium bromide were barely biodegradable. In the case of two of them, no biotransformation products were detected. The elongation of the alkyl side chain made the IL more susceptible for microbiological decomposition. 1-Decyl-3-methyl-imidazolium bromide was biotransformed most easily. Its primary biodegradation up to 100 % could be achieved. Nevertheless, the cleavage of the imidazolium ring has not been observed.

**Electronic supplementary material:**

The online version of this article (doi:10.1007/s10532-015-9747-0) contains supplementary material, which is available to authorized users.

## Introduction

Ionic liquids, because of their unique physicochemical properties, are a promising group of chemicals that can be widely used in various branches of industry. These compounds have therefore been investigated intensively for the last two decades with regard to their biological properties and their effect on the environment (Jastorff et al. 2003; Gathergood et al. 2006; Stolte et al. 2008; Coleman and Gathergood 2010; Pham et al. 2010; Siedlecka et al. 2011; Markiewicz et al. 2013). This research comprised two main areas: ecotoxicity and biodegradability of ionic liquids. In order to evaluate biodegradability of ILs, standard OECD tests were usually applied. They revealed that many of the ionic liquids were not susceptible to biological decomposition. Particularly imidazolium ionic liquids attracted the most interest in industry and academia. Standard biodegradation tests focused mainly on primary biodegradation, which is an alteration in the chemical structure of a substance, by the biological action, resulting in the loss of the specific property of that substance (OECD 2014).

Stolte et al. (2008) tested the primary biodegradation of different *N*-imidazoles, imidazolium, pyridinium and 4-(dimethylamino)pyridinium compounds substituted with various alkyl side chains and their functionalized analogues. Significant primary biodegradation (up to 100 %) for the ionic liquids carrying long alkyl side chains (C6 and C8) was noticed, whereas in the case of imidazolium ILs with short alkyl (≤C6) and short functionalized side chains, no biological decomposition was found. Docherty et al. (2007) showed that hexyl- and octyl-substituted pyridinium ILs could be totally metabolized; the imidazolium analogues were partially mineralized, whereas butyl-substituted imidazolium ILs were not biodegradable. The results of biodegradation tests indicated that a certain lipophilicity of ionic liquids was required to increase their biodegradability (Docherty et al. 2007; Stolte et al. 2008; Pham et al. 2010). It was also observed that the introduction of various functional groups into the side chain (e.g. terminal hydroxyl, carboxyl, ether and nitrile group) did not lead to the expected improvement of the biodegradation of imidazolium compounds (Stolte et al. 2008). In the case of other cations, the introduction of hydroxyl groups made the ionic liquids more biodegradable (Neumann et al. 2014).

Previous studies on the biodegradation of ionic liquids were usually made with mixed cultures of microorganisms (including flocculent and granular activated sludge). Also, the effect of ionic liquids on flocs morphology and metabolic activity of microorganisms was estimated. Anaerobic granular sludge occurred to be less sensitive to pyridinium-based as well as imidazolium-based ionic liquids than the aerobic sludge (Gotvajn et al. 2014). At low concentration (up to 5 mg l^−1^), imidazolium ionic liquids did not act on the morphology of the flocculent activated sludge, whereas at higher concentrations, they contributed to the decrease of the projected area of sludge flocs (Gendaszewska and Liwarska-Bizukojc 2013). The inhibitory effect on dehydrogenase activity of activated sludge biomass increased with the increase in chain length of the alkyl substituent; however, it was dependent on the origin and properties of activated sludge (Liwarska-Bizukojc 2011). Markiewicz et al. (2009) estimated that at 1-methyl-3-octyl-imidazolium chloride concentration higher than 0.2 mM, the dehydrogenase activity of the cells dropped markedly. Also, Azimova et al. (2009) measured the effect of imidazolium-derived ionic liquids on bacterial respiration rate. It occurred that the values of effect concentration (EC50) were similar to those for 1-butanol, which is the alcohol with the alkyl chain length similar to that of the cation of the tested compound like, for example, 1-butyl-3-methyl-imidazolium bromide (Azimova et al. 2009).

Apart from the mixed cultures of activated sludge, the pure cultures of bacteria or the isolated consortia were employed in the biodegradation of ionic liquids, too. Abrusci et al. (2011) found that more than half of 37 studied ionic liquids exhibited biodegradation percentage greater or equal than 60 % after a 28-day incubation with the bacterium *Sphingomonas paucimobilis* at 45 °C. Megaw et al. (2013) identified the bacterial isolates, out of which two were particularly effective ionic liquid biodegraders and were regarded as the candidates for the bioremediation of 1-ethyl- and 1-butyl-3-methyl-imidazolium chlorides. At the same time, biodegradation of 1-methyl-3-octyl-imidazolium chloride ([OMIM][Cl]) conducted by the isolated consortium of bacteria was lower than that performed with the use of activated sludge organisms (Markiewicz et al. 2014). It might have been a result of lower cell densities in the samples with the isolated consortium (Markiewicz et al. 2014).

The next step in the studies on biodegradability of ILs was to check the ability of microorganisms to be adapted to the presence of ionic liquids and finally to use them as a carbon source. Markiewicz et al. (2011) observed a nearly 30-fold increase of the biodegradation rate of 1-methyl-3-octyl-imidazolium chloride ([OMIM][Cl]) during the process of adaptation of activated sludge. At the same time, the supplementation with organic carbon and nitrogen decreased biodegradation rate of this IL (Markiewicz et al. 2011). The results presented by Romero et al. (2008) revealed that 1-alkyl-3-methyl-imidazolium chlorides were poorly biodegradable even if the additional carbon source, in this case glucose, was available. During ten days of aerobic biodegradation, glucose was totally consumed, whereas the concentration of ionic liquids decreased only slightly below the initial value 100 mg l^−1^. On the contrary, Gotvajn et al. (2014) observed the appearance of co-metabolism in the biodegradation of ionic liquids, when glucose was added.

In order to describe biodegradation of any compounds precisely, it is essential to identify the intermediates. The latter was performed using most often liquid chromatographic methods coupled to mass spectrometry including ion trap mass spectrometer (Stolte et al. 2008; Pham et al. 2009; Coleman and Gathergood 2010; Markiewicz et al. 2011; Neumann et al. 2014). In some cases, ^1^H nuclear magnetic resonance (NMR) analyses can be used (Deng et al. 2011). Nevertheless, the number of publications concerning metabolites of ILs biodegradation and suggestion of their biodegradation pathways is very limited. For 1-methyl-3-octyl-imidazolium cation, different biological transformation products carrying hydroxyl, carboxyl and carbonyl groups were identified (Stolte et al. 2008). This ionic liquid and its hydroxylated and carboxylated analogues were completely degraded if primary biodegradation is considered. At the same time, Pham et al. (2009) observed that biodegradation of 1-butyl-3-methylpyridinium bromide led to the formation of 1-hydroxybutyl-3-methylpyridine, 1-(2-hydroxybutyl)-3-methylpyridine, 1-(2-hydroxyethyl)-3-methylpyridine and methylpyridine. Any product of further degradation, including intermediates of pyridinium ring cleavage, was not found. Markiewicz et al. (2011) proposed that the biodegradation of 1-methyl-3-octyl-imidazolium cation started with the ω-oxidation of the alkyl chain, and this chain was subsequently degraded via β-oxidation. They observed ultimate degradation of the imidazolium ionic liquid [OMIM][Cl]; however, any degradation products of imidazolium ring cleavage were neither shown nor listed (Markiewicz et al. 2011). Neumann et al. (2014) found that imidazolium ionic liquids were the most refractory out of five cation groups tested. No biodegradation was observed for the imidazolium ionic liquid with propyl side chains (Neumann et al. 2014).

The main aim of this work was to identify the products of biodegradation of seven imidazolium ionic liquids, including three conventional 1-alkyl-3-methyl-imidazolium bromides and four novel, recently synthesized peralkylated ILs. These novel imidazolium ionic liquids to be tested in this work possess the improved physicochemical properties compared to the conventional imidazolium ionic liquids (Maton et al. 2012). However, the knowledge about their microbiological decomposition and potential effect on the environment, particularly its aquatic compartment, is limited. Based upon the identified metabolites, the most probable biodegradation pathways of the studied ILs were proposed.

## Materials and methods

### Tested compounds

Seven ionic liquids of different chemical structures were tested. Three of them were 1-alkyl-3-methyl-imidazolium bromides with an increasing alkyl chain length. They were purchased from Ionic Liquids Technologies GmbH (Denzlingen, Germany). The other four ionic liquids were synthesized by the Department of Sustainable Organic Chemistry and Technology (Ghent University, Belgium). These were tetra- or completely substituted imidazolium iodides. The short description of the ionic liquids including their chemical composition and monoisotopic molecular weight of their cations is presented in Table 1.

**Table 1:** Chemical names, codes and molecular weights of ionic liquids tested

Chemical name	Code	Elemental composition	Average molecular weight of IL (g mol^−1^)	Monoisotopic molecular weight of cation (g mol^−1^)
1-Ethyl-3-methyl-imidazolium bromide	IL1	C_6_H_11_N_2_Br	191.1	111.0945 (+0.0023)
1-Hexyl-3-methyl-imidazolium bromide	IL2	C_10_H_19-_N_2_Br	247.2	167.1554 (+0.0006)
1-Decyl-3-methyl-imidazolium bromide	IL3	C_14_H_27_N_2_Br	303.3	223.2154 (+0.0020)
1-Ethyl-2-isopropyl-3-methyl-4,5-dimethylimidazolium iodide	IL4	C_11_H_21_N_2_I	308	181.1715 (+0.00010)
1-Ethyl-2-methyl-3-methyl-4,5-dimethyl-imidazolium iodide	IL5	C_9_H_17_N_2_I	280	153.1401 (+0.00010)
1-Ethyl-2*H*-3-methyl-4,5-dimethylimidazolium iodide	IL6	C_8_H_15_N_2_I	266	139.1245 (+0.0010)
1-Hexyl-2*H*-3-methyl-4,5-dimethylimidazolium iodide	IL7	C_12_H_23_N_2_I	322	195.1856 (+0.0006)

### Biodegradation tests

In the tests, activated sludge taken from the aeration chamber of the municipal wastewater treatment plant in Zgierz (Poland) was used as inoculum. The sludge was subjected to sedimentation, then the supernatant was discarded and solid particles were resuspended in synthetic wastewater, which contained 300 mg peptone, 100 mg sodium acetate, 50 mg potassium monophosphate, 50 mg sodium bicarbonate, 50 mg ammonium hydrophosphate, 5 mg magnesium sulphate and 5 mg sodium chloride per litre. It was repeated twice in order to ensure removal of any undesired contaminants that could be present in the activated sludge suspension and might influence the biodegradation process of the ionic liquids. Preparation of the inoculum was made in agreement with the standards presented in the guidelines of OECD 301A test. A 40 ml of activated sludge (inoculum) was transferred into a 300-ml Erlenmeyer flask, containing 160 ml of fresh synthetic wastewater with or without imidazolium ionic liquid. The composition of the synthetic wastewater was the same as above. At the beginning of the test, the biomass concentration expressed as volatile suspended solids (VSS) was 545 ± 14 mg l^−1^, while the concentration of COD and BOD was 645 ± 30 mg O_2_ l^−1^ and 395 ± 12.4, respectively. Each ionic liquid was tested separately at an initial concentration of 50 mg l^−1^. The control run without any ionic liquids was performed in parallel. Also sterile controls for each ionic liquid tested were made. The flasks were incubated at 20 ± 0.1 °C in a thermostated rotary shaker Certomat^®^ IS at the rotation speed 130 min^−1^ for 21 days. Each flask was supplemented with a small amount (10 ml) of synthetic wastewater twice a week to elevate COD up to 300 ± 6 mg O_2_ l^−1^. This was added in order to ensure the appropriate amount of easily available carbon, nitrogen and microelements to the activated sludge. The experiments for all ionic liquids and the controls were made in duplicate.

### Analysis of ionic liquids and their metabolites

The concentration of the ILs was determined with the use of UPLC^®^ (Waters, USA). A Waters Symmetry Shield RP18 column was used at various CH_3_CN–H_2_O (v/v) isocratic elutions (both eluents modified with 1 % of formic acid): 2:98, 15:85 and 40:60 for IL1, IL2 and IL3, respectively. For the other four ILs, the compositions of the eluents were as follows: 2:98 (v/v) for IL4 and IL5, 10:90 (v/v) for IL6 and 20:80 (v/v) for IL7. The temperature of the column was 40 °C. All ionic liquids, namely their cations, were detected by a Synapt G2 MS–MS detector at the positive electrospray ionization (ESI+) with the use of the following M+, ion masses: 111.0945, 167.1554, 223.2154, 181.1724, 153.1401, 139.1245 and 195.1856 for IL1, IL2, IL3, IL4, IL5, IL6 and IL7, respectively. The settings of the mass detector in all analyses were as follows: capillary voltage 3 kV, sampling cone voltage 40 V, extraction cone voltage 4 V, source temperature 120 °C, desolvation temperature 200 °C and desolvation gas (nitrogen) flow rate 500 l h^−1^.

For the detection of the products of the biotransformation, mass spectrometry with the positive electrospray ionization was used in the TOF mode. The positive ions were also detected in the form of M+, i.e. no hydrogen atom was attached to the detected molecule during the analysis. All formulae and molecular masses mentioned below are given for the cation only. The anions of the ionic liquid were disregarded in this analysis, as they became neutral after ionization and were not observed by the detector. The limit of the detection was estimated at 0.01 mg l^−1^. It allowed for the appropriate identification of the imidazolium ions and their biodegradation products.

### Other analyses

At the beginning and at the end of each test, the following analyses were performed: soluble chemical oxygen demand (COD), biochemical oxygen demand (BOD_5_), total solids (TS), total suspended solids (TSS), volatile solids (VS) and volatile suspended solids (VSS) in agreement with the standard procedures (APHA-AWWA-WEF 2012).

## Results and discussion

First of all, none of the ILs studied was completely biodegradable to inorganic products such as carbon dioxide and water. This confirms previous experiments using OECD tests (Liwarska-Bizukojc and Gendaszewska 2013; Liwarska-Bizukojc et al. 2014). Thus, the term biodegradation of ionic liquids has been substituted by the term biotransformation or primary biodegradation in order to be more precise in the description below.

**Fig. 1:**
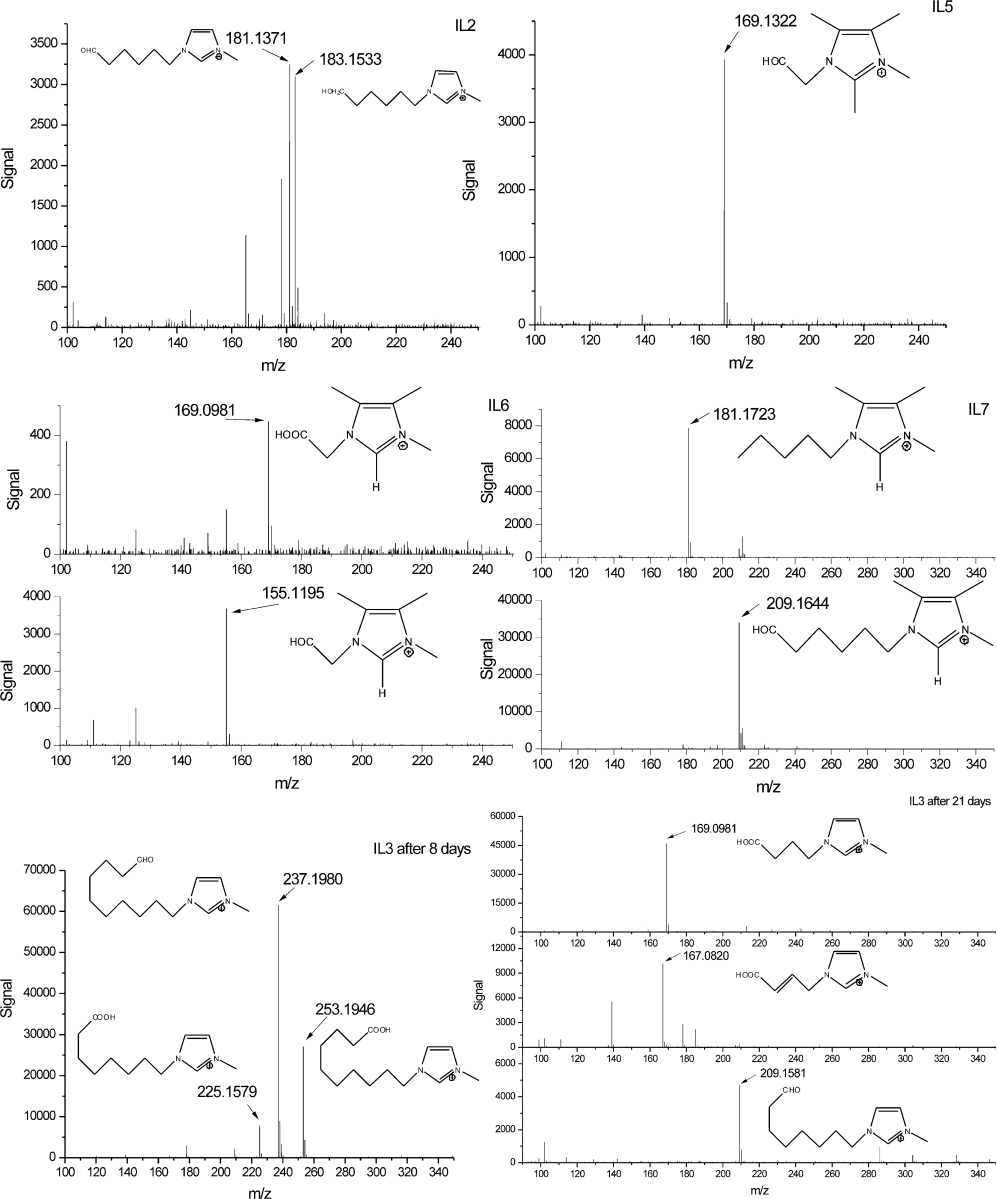
Mass spectra of the detected degradation intermediates of imidazolium ionic liquids tested

In Table 2, the identified metabolites of the biotransformation of the imidazolium ionic liquids tested are presented. Their mass spectra are depicted in Fig. 1. It occurred that two of the seven ionic liquids did not show any biotransformation. These were 1-ethyl-3-methyl-imidazolium bromide (IL1) and 1-ethyl-2-isopropyl-3-methyl-4,5-dimethylimidazolium iodide (IL4). In both cases, the alkyl substituents were short and did not contain more than three carbon atoms. This is in agreement with earlier observations proving that ionic liquids with short alkyl side chains (≤C6) were poorly or even not biodegradable (Docherty et al. 2007; Liwarska-Bizukojc and Gendaszewska 2013; Liwarska-Bizukojc et al. 2014). In addition, the results of this work indicated that it was independent of the number of alkyl substituents on the imidazolium ring.

**Table 2:** Ionic liquids and their biotransformation products

**Fig. 2:**
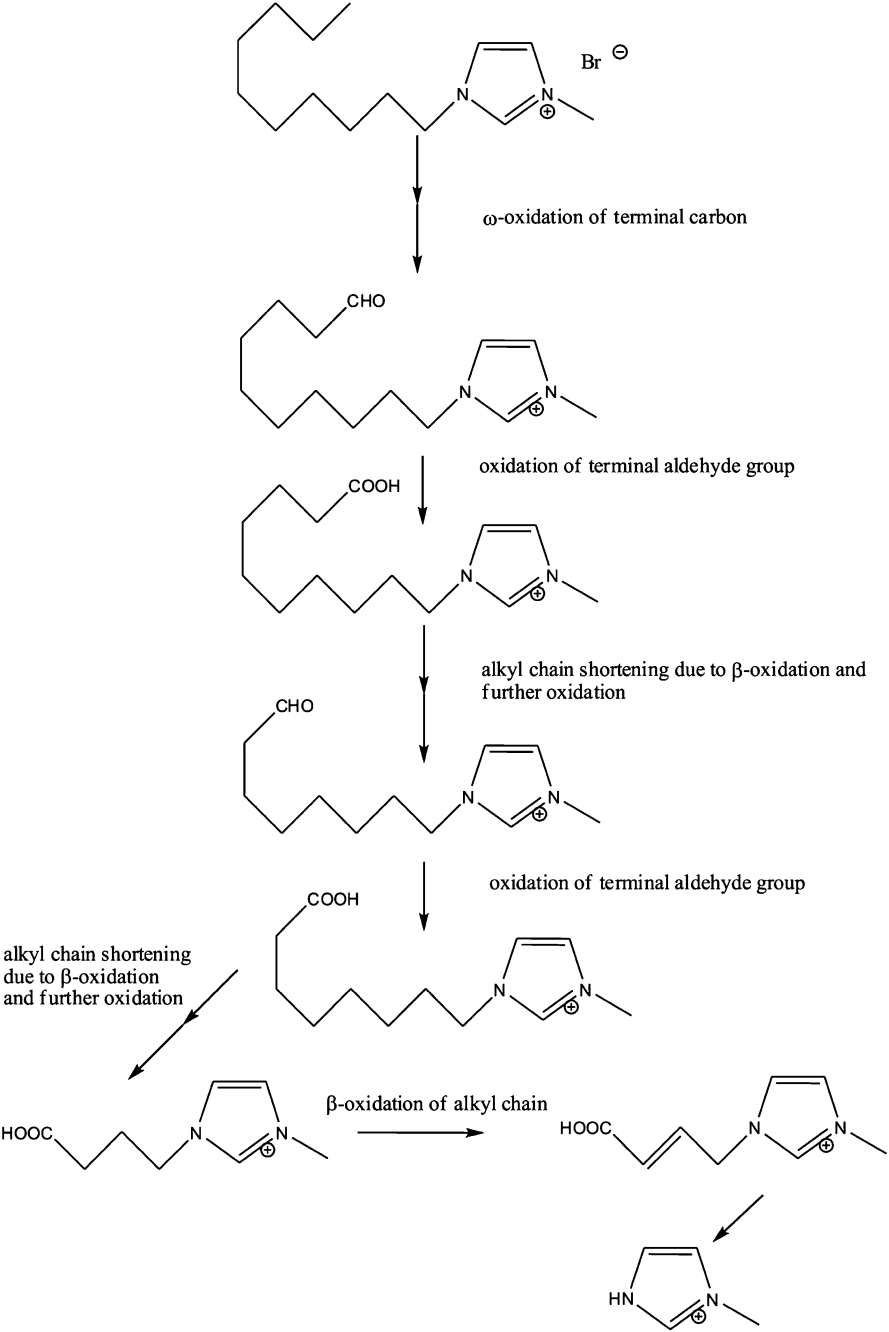
Most probable biodegradation pathway of IL3

At the same time, biotransformation was much further advanced for the ionic liquid having the longest alkyl chain, i.e. 1-decyl-3-methyl-imidazolium bromide (IL3). In the case of IL3, a variety of products of biotransformation were identified. These were carboxylic acids, aldehydes and unsaturated carboxylic acids (Table 2; Fig. 1). The composition of these products varied also in time, which proved the progress of the biotransformation processes of the molecule studied. The detected metabolites of IL3 indicated that the decyl side chain was hypothetically oxidized via ω-oxidation catalysed by monooxygenase and then, subsequently, via β-oxidation leading to formation of the butyl side chain after 21 days of biodegradation. In Fig. 2, the most probable metabolic pathway of the ionic liquid IL3 was proposed. It is suggested that the 3-methyl-imidazolium ion remains intact after oxidation of the decyl side chain of IL3 (Fig. 2) and that this ion is not susceptible for microbiological decomposition. This was also confirmed using EAWAG-BBD Pathway Prediction System (http://eawag-bbd.ethz.ch/predict/) and the module PathPred (http://www.genome.jp/tools/pathpred/) of Kyoto University Bioinformatics Center (Online Resource 1). The proposed pathway was consistent with the findings presented by Kumar et al. (2006) and the theoretical metabolic scheme elaborated by Jastorff et al. (2003). On the contrary to these observations, Markiewicz et al. (2011) stated full degradation of the imidazolium ring; however, its degradation products were not shown in this work. At the same time, the cleavage of the imidazolium ring of the ionic liquids was successfully achieved with the help of advanced oxidation methods such as the Fenton-like-reaction or the electrochemical degradation (Siedlecka et al. 2011). Borek and Waelsch (1953) found intermediates of l-histidine (protein amino acid) biodegradation indicating that enzymatic cleavage of the imidazolium ring was possible. Nevertheless, the chemical structure of l-histidine is quite different from the ionic liquids tested here. Regarding metabolites of decomposition of imidazolium ionic liquids known so far and the results of using of bioinformatics tools to predict biodegradation pathways of the studied compounds, it was found that the imidazolium ring was a stable structure, and there were no evidences that any bacterial enzymes were able to destroy it.

Two breakdown products of primary biodegradation of IL2 were detected. These were the ions of *m*/*z* = 183.1533 [Δ(*m*/*z*) = +0.0036] and *m*/*z* = 181.1371 [Δ(*m*/*z*) = +0.0031]. The first one was the primary alcohol since the difference in masses between IL2 ion and this ion was 15.9979 (mass of an oxygen atom) and indicated the transformation of the –CH_3_ moiety into –CH_2_OH (C_10_H_19_ON_2_). The second one is an aldehyde moiety formed by the oxidation of the aforementioned alcohol. The difference in masses between IL2 ion and this ion is 13.9817, which indicates the addition of one oxygen atom and the removal of two hydrogen atoms: –CH_3_ vs. –CHO (C_10_H_17_ON_2_). After 21 days of the process, only the aforementioned aldehyde was detected. No signs of an ion that could be attributed to the analogous alcohol were found. In the case of IL2, the longest alkyl, i.e. hexyl chain, was terminally oxidized.

With regard to the completely substituted ionic liquid, 1-ethyl-2-methyl-3-methyl-4,5-dimethylimidazolium iodide (IL5), one product of biotransformation was identified. Independent of the process duration, the same ion of *m*/*z* = 169.1322 [Δ(*m*/*z*) = −0.0020] (Table 2; Fig. 1) was detected. It clearly indicated the addition of a single oxygen atom to the ionic liquid molecule (–CH_3_ transformed into –CH_2_OH), namely the formation of the primary alcohol (C_9_H_17_ON_2_) (Fig. 1). However, further biodegradation of this ionic liquid was stopped (or at least slowed down) most probably due to the short side chains, i.e. methyl or ethyl substituents. It is worth noticing that the second fully substituted ionic liquid tested in this work (IL4) did not show any biotransformation products despite the fact that one of side chains was longer (i.e. isopropyl) than those of IL5 (Table 2). These observations indicated that not only the length but also the chemical structure of the alkyl substituent (for example branched alkyl groups) influenced the bioavailability of the molecule.

The ionic liquid IL6 is a tetrasubstituted compound of the same chemical structure as IL5 excluding the presence of a methyl group at position C2 (Table 1). However, in the case of biotransformation of IL6, not only the primary alcohol but also the carboxylic acid was found (Table 2; Fig. 1). The latter was formed as a result of oxidation of the alcoholic moiety and was detected only after 21 days of biodegradation. It was illustrative for the progress of biotransformation of IL6.

With regard to the tetrasubstituted ionic liquid IL7, two products of biotransformation were identified. These were the ions of *m*/*z* = 209.1644 [Δ (*m*/*z*) = +0.001] and *m*/*z* = 181.1723 [Δ (*m*/*z*) = +0.0018] (Table 2; Fig. 1). The first one was 13.9793 larger than IL7 ion, which indicates the transformation of the –CH_3_ moiety into –COH (one oxygen atom added and two hydrogen atoms removed) and formation of an aldehyde. At the same time, the mass of the second identified ion was smaller than that of IL7 ion and represented the ion of the formula C_12_H_21_N_2_. This was the same ionic liquid cation, but with a pentyl side chain instead of a hexyl side chain. Both metabolites were found after 8 as well as 21 days of process. It suggested that biodegradation of IL7 slowed down after 8 days.

**Fig. 3:**
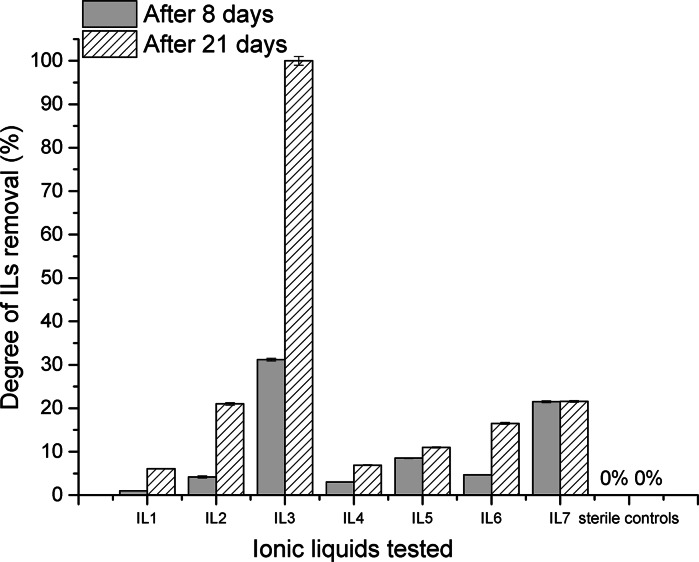
Degrees of removal of the ionic liquids tested dependent on the duration of the process; the *error bars* reflect the variation of removal of each ionic liquid in the duplicated biodegradation tests

In Fig. 3, the degree of removal of the ionic liquids is depicted. They were calculated on the basis of the determination of the monoisotopic cation concentration. This determination is of high repeatability and the values of standard deviation for the replicated analysis did not exceed 1.7 %. It was assumed that not only biodegradation, but also other physicochemical processes like sorption were involved in the removal of ionic liquids from wastewater. However, the sorption of organic pollutants on activated sludge was usually faster than biodegradation and it is regarded as the instantaneous process, difficult to be unequivocally expressed quantitatively (Pomiès et al. 2013). Beaulieu et al. (2008) showed that alkylmethylimidazolium-based ILs did not strongly adsorb to the tested aquatic sediments suggesting that sorption was not the main mechanism in the removal of this type of ionic liquids. In general, the degrees of removal of ionic liquids from wastewater were consistent with the aforementioned results of this work. First of all, no removal of ionic liquids was observed in the sterile controls (Fig. 3). The highest degree of removal equal to 100 % after 21 days was found for IL3. Then, the degrees of 21 and 22 % for IL2 and IL7 were achieved, respectively. For the other ionic liquids studied here, the degrees of removal were low and did not exceed 16.4 % after 21 days (Fig. 3). The lowest values were found for both completely substituted ionic liquids IL4 and IL5 and for 1-ethyl-3-methyl-imidazolium bromide (IL1) (Fig. 3). Taking the following fact into account that for IL1 and IL4 any biodegradation products were not found, their removal (below 5 %) may have been the result of sorption processes or alternatively of the very initial microbiological decomposition, which ran to such a small extent that the biodegradation products could not be detected despite the application of high-resolution techniques.

## Conclusions

1. The following hypothetical biotransformation pathway for imidazolium ionic liquids can be proposed. The terminal carbon atom of the longest substituent is subjected to oxidation catalysed by monooxygenases (formation of the primary alcohol); next this alcohol moiety is oxidized by dehydrogenases to an aldehyde and carboxylic acid moiety. Finally, the carboxylic acid is subjected to further oxidation by β-oxidation, and as a result, shorter side chains are formed. The fact that sometimes only one carbon atom is removed suggests that it is not β-oxidation but simple decarboxylation. On the other hand, the presence of an unsaturated intermediate in the side chain (in the case of IL3) is an indirect evidence for β-oxidation either. Both mechanisms are probably active.
2. Longer alkyl side chains are more susceptible to oxidation of the terminal carbon atom. IL3, having a decyl side chain, is the most easily biotransformed of the ILs studied. Nevertheless, no products of cleavage of the imidazolium ring were observed.
3. The increase of the number of substituents of the imidazolium ring does not favour biodegradation of the ionic liquids. The completely substituted ionic liquids were less susceptible to biodegradation than the ionic liquids with a lower number of alkyl side chains. Ionic liquids, which are completely substituted by short alkyl chains (up to two carbon atoms), are the most difficult to be decomposed microbiologically. It should be taken into account when the synthesis of environment-friendly imidazolium ionic liquids will be designed in the future.

## Electronic supplementary material

Supplementary material 1 (DOC 138 kb)
