# Supplementary material for: Biodegradation of imidazolium ionic liquids by activated sludge microorganisms
Source: Biodegradation. 2015 Oct 13;26(6):453–63. doi: 10.1007/s10532-015-9747-0 (PMC4637002; doi:10.1007/s10532-015-9747-0)

Biodegradation of imidazolium ionic liquids by activated sludge microorganisms

Biodegradation

Ewa Liwarska-Bizukojc^*^, Cedric Maton, Christian V. Stevens

^*^Lodz University of Technology, Institute of Fermentation Technology and Microbiology, ul. Wolczanska 171/173, 90-924 Lodz, Poland, phone +48 42 636 36 39, fax +48 42 636 59 76, e-mail: ewa.liwarska-bizukojc@p.lodz.pl


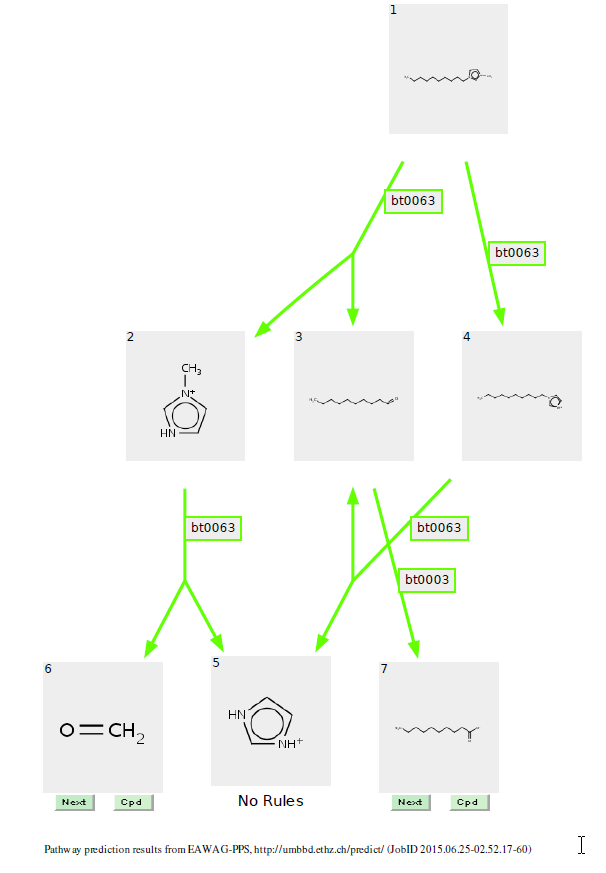


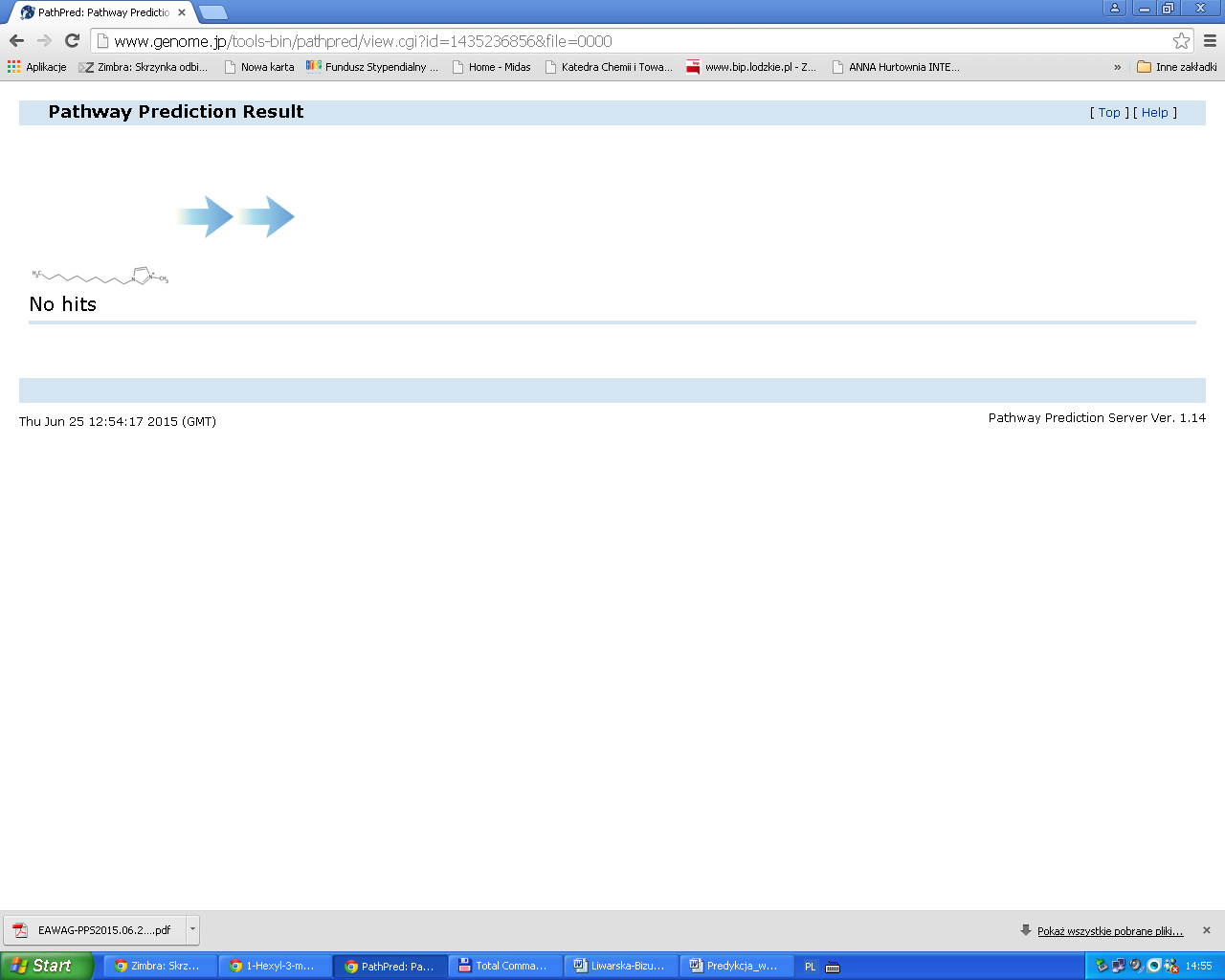

Supplement: Supplementary file 1 — Supplementary material 1 (DOC 138 kb) [file 10532_2015_9747_MOESM1_ESM.doc]
